# Supplementary material for: BiPOLES is an optogenetic tool developed for bidirectional dual-color control of neurons
Source: Nat Commun. 2021 Jul 26;12:4527. doi: 10.1038/s41467-021-24759-5 (PMC8313717; doi:10.1038/s41467-021-24759-5)
Supplement: Supplementary file 1 — Supplementary Information [file 41467_2021_24759_MOESM1_ESM.pdf]

BiPOLES is an optogenetic tool developed for bidirectional dual-color control of neurons

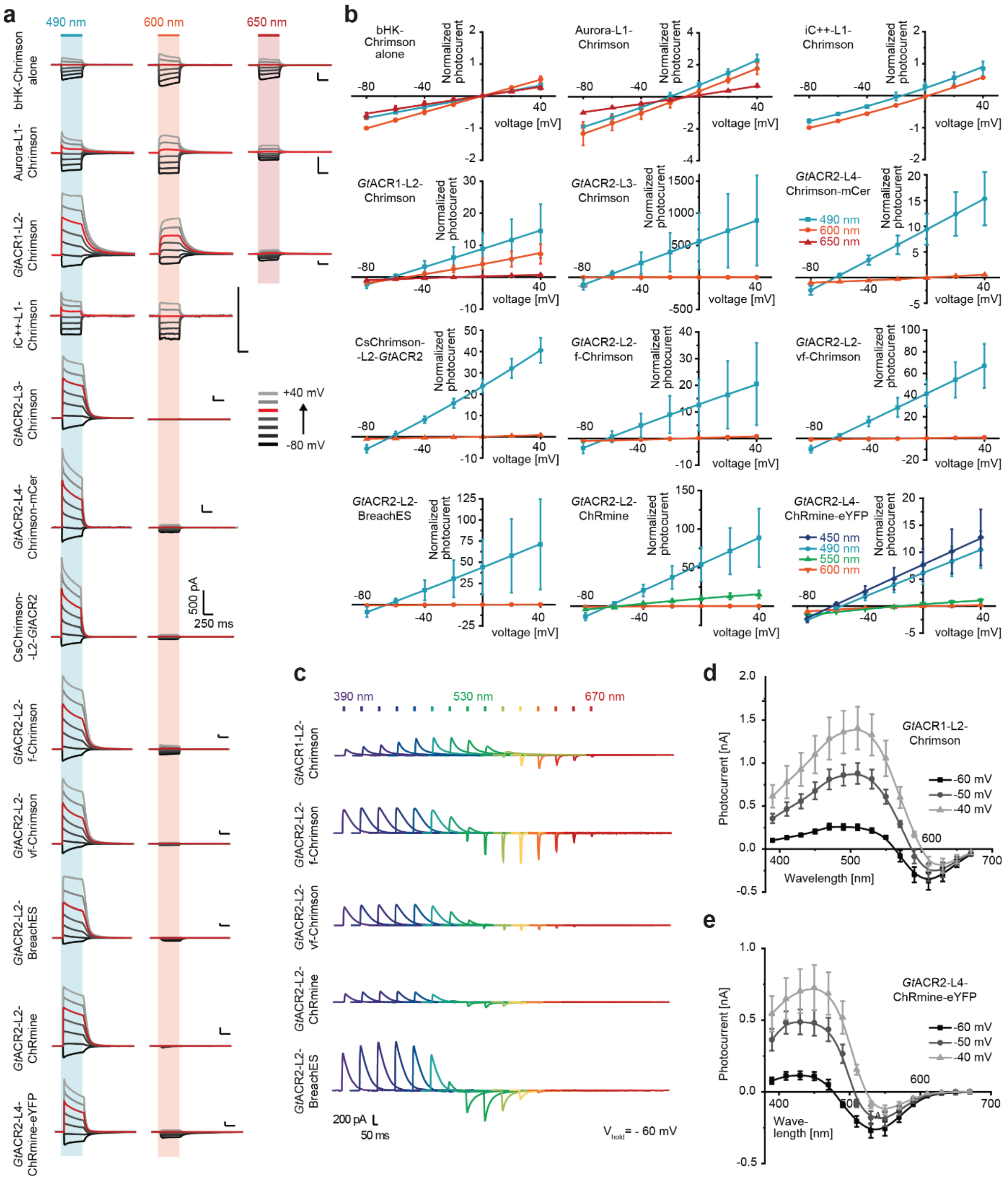

Supplementary Fig. 1. Biophysical characterization of different ACR-CCR tandem constructs. (a) Representative photocurrents of ßHK-Chrimson and different tandem constructs as described in Fig. 1a. **(b)** Normalized peak photocurrents of ßHK-Chrimson and tandem constructs at different membrane voltages evoked at 450 nm, 490 nm, 530 nm or 600 nm (see panel (a), mean ± SD; n indicates number of independent cells. n = 4 for ßHK-Chrimson; n = 5 for Aurora-L1-Chrimson, *Cs*Chrimson-L2-*Gt*ACR2 and *Gt*ACR2-L2-f-Chrimson; n = 6 for *Gt*ACR2, *Gt*ACR1-L2-Chrimson and *Gt*ACR2-L2-vf-Chrimson; n = 7 for iC++-L1-Chrimson, *Gt*ACR2-L3-Chrimson, *Gt*ACR2-L4-Chrimson-mCer, *Gt*ACR2-L2-BreachES and *Gt*ACR2-L2-ChRmine; n = 8 for *Gt*ACR2-L2-Chrimson and n = 9 for *Gt*ACR2-L4-ChRmine-ts-eYFP-er); normalized to the peak photocurrent at -80 mV and 600 nm illumination). **(c)** Representative photocurrents of different ACR-CCR tandems with 10 ms light pulses at indicated wavelengths and equal photon flux at -60 mV. **(d, e)** Action spectra of *Gt*ACR1-L2-Chrimson and *Gt*ACR2-L4-ChRmine-

TS-eYFP-ER at different membrane voltages (mean ± SEM, n = 6 for *Gt*ACR1-L2-Chrimson and n = 8 for *Gt*ACR2-L4-ChRmine-TS-eYFP-ER). The data presented in this figure are provided in the Source Data file.

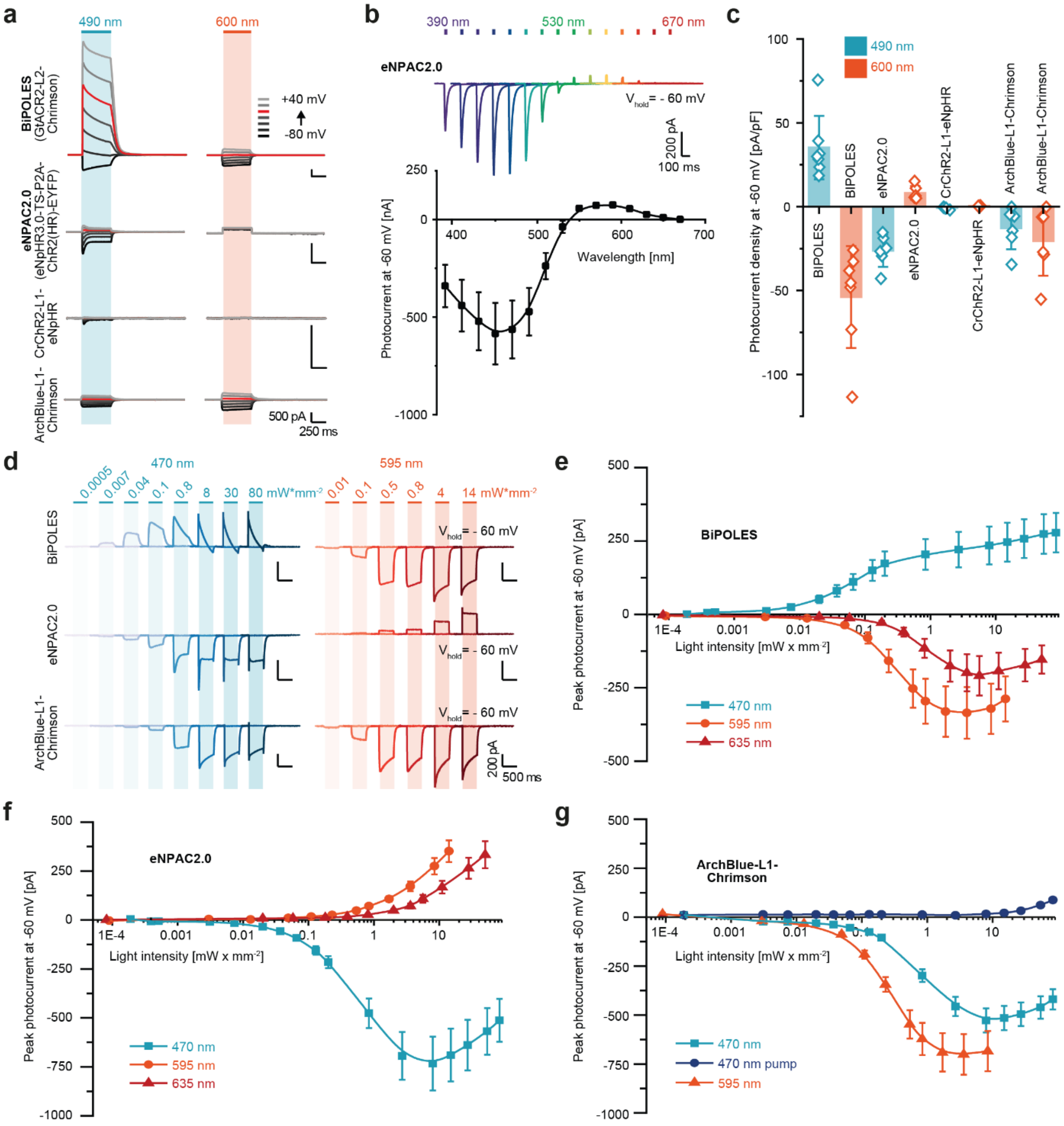

Supplementary Fig. 2. Comparison of BiPOLES to established bidirectional optogenetic tools in HEK293 cells. (a) From top to bottom: representative photocurrents of BiPOLES, eNPAC2.0 (eNpHR3.0-TS-p2A-*Cr*ChR2(H134R)-EYFP), *Cr*ChR2-L1-eNpHR[2] and ArchBlue-L1-Chrimson in whole-cell patch clamp recordings from HEK293 cells at 490 nm and 600 nm illumination. ArchBlue stands for the blue shifted mutant of Arch3.0[26]. **(b)** Top: Representative photocurrents of eNPAC2.0 with 10 ms light pulses at indicated wavelengths and equal photon flux at -60 mV. Bottom: Action spectrum of eNPAC2.0 at -60 mV (mean ± SEM, n = 5). **(c)** Peak photocurrent densities for 490 nm and 600 nm excitation at -60 mV (close to the neuronal resting potential) as shown in (a) (Mean ± SD; n indicates number of independent cells. n = 5 for *Cr*ChR2-L1-NpHR; n = 6 for ArchBlue-L1-Chrimson and eNPAC2.0, n = 7 for BiPOLES). **(d)** Representative photocurrents of BiPOLES (top), eNPAC2.0(middle) and ArchBlue-L1-Chrimson (bottom) at -60 mV and different irradiances and wavelengths. **(e-g)** Peak photocurrents at different irradiances, different excitation wavelength and -60 mV according to (d). (mean ± SEM, n = 4 for ArchBlue-L1-Chrimson and n = 6 for BiPOLES and eNPAC2.0) 6). Pump currents at 470 nm in (g) describe the initial outward currents observed directly after blue light switching in (d). The data presented in this figure are provided in the Source Data file.

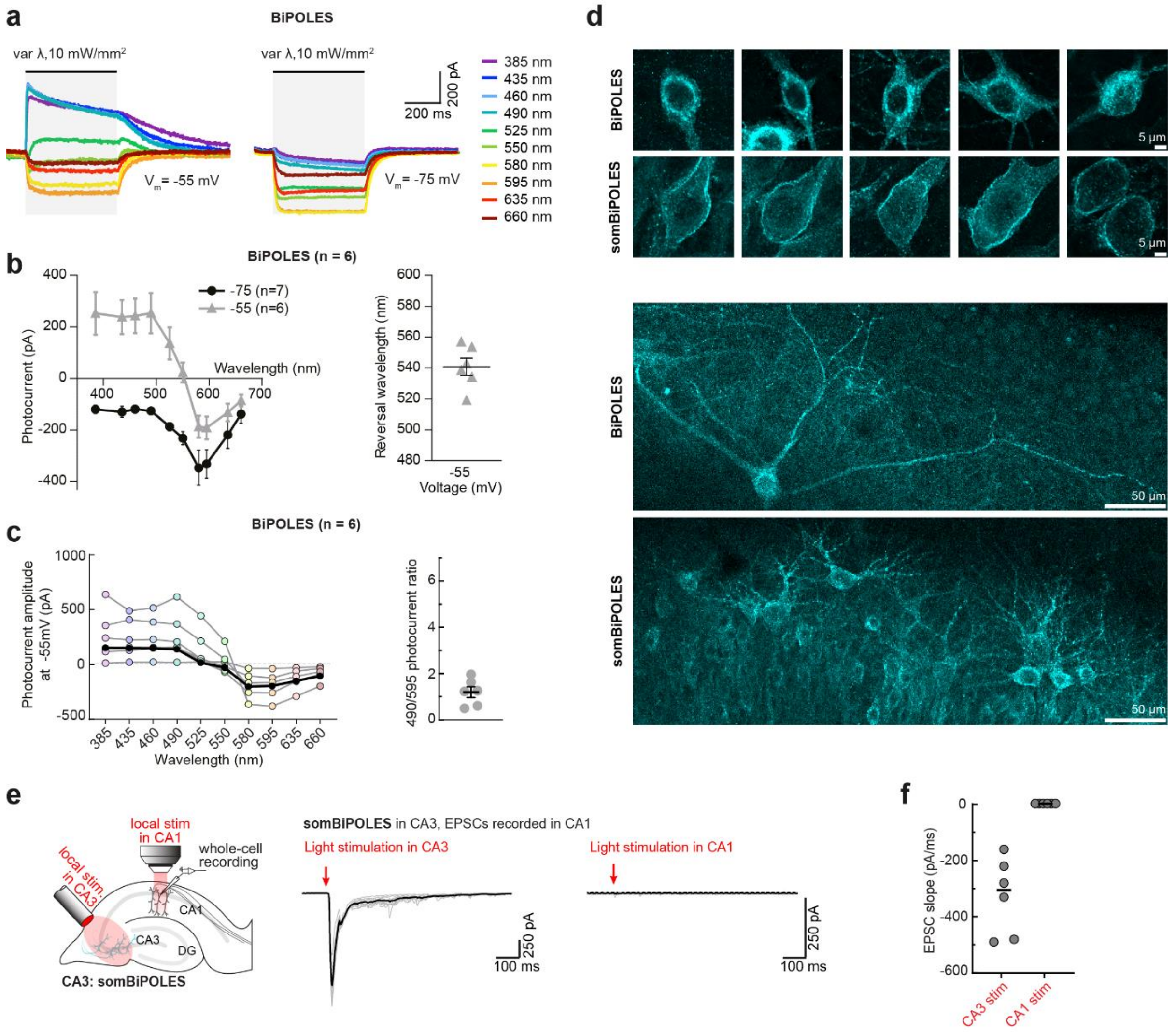

Supplementary Fig. 3. Biophysical characterization of BiPOLES and differential expression of BiPOLES and somBiPOLES in CA1 pyramidal neurons. (a) Representative photocurrent traces of BiPOLES in CA1 pyramidal neurons upon illumination with different wavelengths and equal photon flux at membrane voltages above (left) and below (right) the chloride Nernst potential. **(b)** Left: Quantification of photocurrent amplitude along the spectrum at a membrane voltage of -55 mV (grey) and -75 mV (black). Symbols indicate mean ± SEM and lines are interpolations of data points ($n_{-55\,mV}$ = 6 cells, $n_{-75\,mV}$ = 7 cells). Similar to HEK-cell measurements, inward and outward photocurrents were evoked with 635 nm and 490 nm at a membrane voltage between the chloride and proton Nernst potentials, respectively, indicative of independently evoked Chrimson- and *Gt*ACR2-photocurrents. Right: Quantification of photocurrent reversal wavelength at -55 mV (mean ± SEM, n = 6 cells). **(c)** Left: Quantification of photocurrent amplitudes at -55 mV (same data as in (b) but showing individual data points for each wavelength, black circles: medians, n = 6 cells). Right: Ratio of inhibitory (490 nm) over excitatory (595 nm) photocurrents (mean ± SEM, n = 6 cells). Note that, unlike for eNPAC2.0 (Supplementary Fig. 8a) the photocurrent ratio shows little variability between cells, indicating a reproducible stoichiometry of Chrimson and *Gt*ACR2 currents. **(d)** Maximum-intensity projections of confocal images showing expression of BiPOLES or soma-targeted BiPOLES (somBiPOLES) in CA3 pyramidal neurons of organotypic hippocampal slices. For each opsin 5 representative neurons from 5 organotypic slices are shown (top rows).. Bottom: lower-magnification example images of CA3 neurons in *stratum oriens* show confinement of somBiPOLES to soma and proximal dendrites. These images were not used for quantitative analysis. CA3 cells were transduced with an AAV9 encoding for either BiPOLES or somBiPOLES and fixed after 20 days. Fluorescence was enhanced by an antibody staining against the fluorophore mCerulean. **(e)** Left: Schematic drawing depicting the experiment used to verify

absence of somBiPOLES-expression in axon terminals of CA3 cells. Whole-cell voltage-clamp recordings were done in postsynaptic CA1 cells to determine red-light evoked EPSCs. Illumination was done locally either in CA3 at the somata or in CA1 at axon terminals of somBiPOLES-expressing CA3 cells. Axon stimulation was done in the presence of TTX to avoid antidromic spiking of CA3 cells and 4-AP to inhibit K^+-mediated fast repolarization. Middle: Example voltage-clamp recordings from CA1 cells upon red-light stimulation in CA3. Right: example voltage-clamp recordings from CA1 cells upon red-light stimulation of axon terminals in CA1. Black lines show average response of 10 repetitions (grey lines). **(f)** Quantification of experiment shown in (e) (black lines: medians, no error bars shown, n = 6 cells). The absence of somBiPOLES-mediated EPSCs upon local illumination in CA1, indicates efficient exclusion of somBiPOLES from the axon terminals, despite strong membrane expression in the somatodendritic compartment, which was evident from large EPSCs upon local illumination in CA3. The data presented in this figure are provided in the Source Data file.

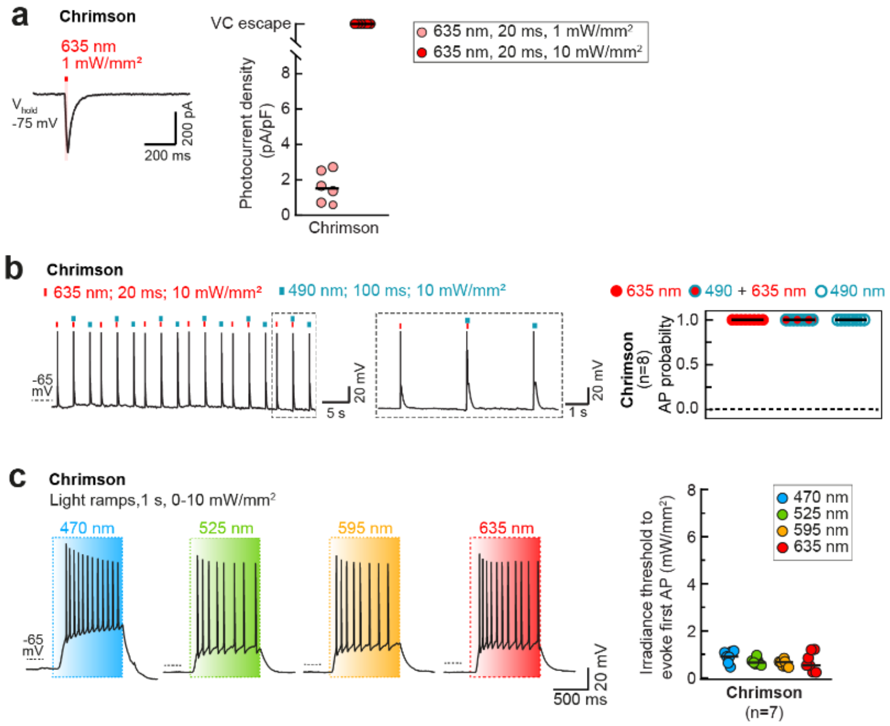

Supplementary Fig. 4. Characterization of Chrimson-mediated currents and spiking in CA1 pyramidal cells. (a) Left: Representative photocurrent trace evoked by a 635 nm light pulse (20 ms, 1 mW mm^{-2}) recorded in a Chrimson-expressing CA1 pyramidal neuron at a membrane voltage of -75 mV. Right: Quantification of photocurrent densities evoked under the indicated conditions (black horizontal lines: medians, n = 6 cells). **(b)** Left: Voltage traces showing red- and blue-light-evoked APs. Right: Quantification of AP probability under indicated conditions (black horizontal lines: medians, n = 8 cells). Note that blue light does not elicit APs in somBiPOLES-expressing cells due to *Gt*ACR2-mediated shunting (see Fig. 4b). **(c)** Spectral quantification of the irradiance threshold for AP generation with Chrimson. Left: Representative membrane voltage traces during light ramps at indicated wavelengths with irradiance increasing linearly from 0 to 10 mW mm^{-2}. Right: Quantification of the irradiance threshold at which the first AP was evoked (black horizontal lines: medians, n = 7 cells). Datasets for 470 and 595 nm are the same as shown in Fig. 3e. The data presented in this figure are provided in the Source Data file.

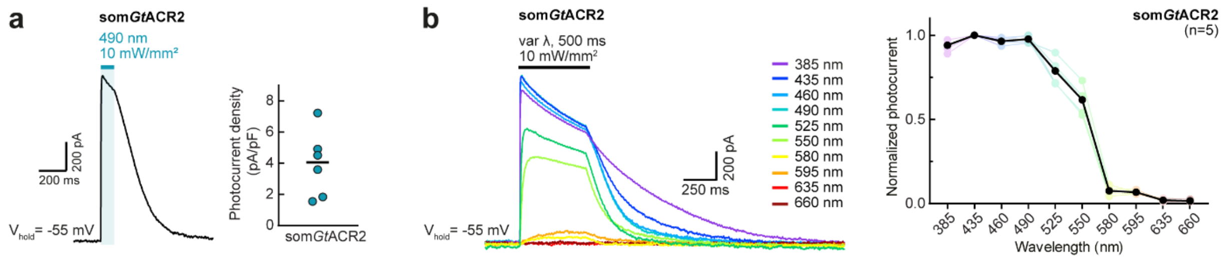

Supplementary Fig. 5. Quantification of som*Gt*ACR2-mediated photocurrents in CA1 pyramidal cells. (a) Left: Representative photocurrent trace evoked by a 490 nm light pulse (100 ms, 10 mW mm^{-2}) recorded in a som*Gt*ACR2-expressing CA1 pyramidal neuron at -55 mV, 20 mV more positive than the chloride Nernst potential. Right: Quantification of photocurrent densities evoked under the indicated conditions (black horizontal lines: medians, n = 6 cells). **(b)** Left: Representative photocurrent traces upon illumination with different wavelengths and equal photon flux at a membrane voltage of -55 mV. Right: Normalized photocurrent amplitude along the spectrum (black circles: medians, n = 5 cells). The data presented in this figure are provided in the Source Data file.

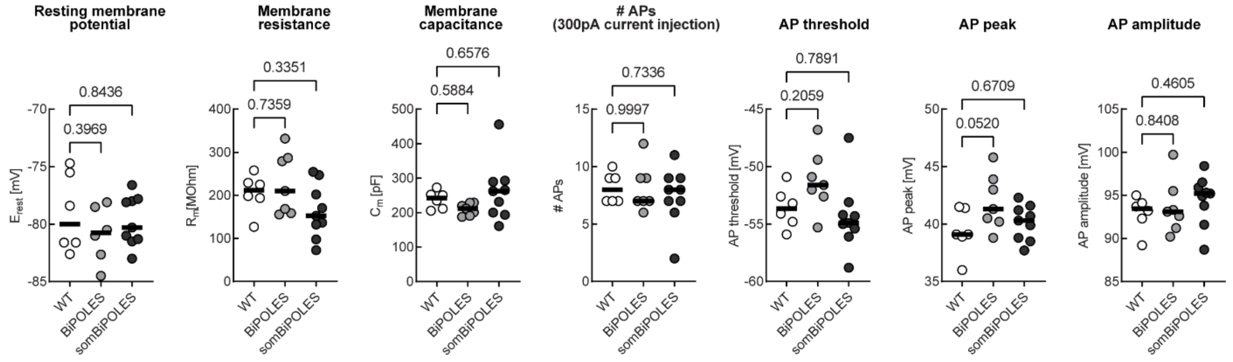

Supplementary Fig. 6: Basic neuronal parameters of WT, BiPOLES- and somBiPOLES-expressing CA1 pyramidal cells. The following parameters were measured to asses cell viability and tolerability of BiPOLES and somBiPOLES: resting membrane potential, membrane resistance, membrane capacitance, number of APs evoked by somatic current injection (300 pA, 500 ms), voltage threshold, peak voltage and AP amplitude of the 1st AP elicited by somatic current injection (black lines: medians, WT n = 6 cells, BiPOLES n = 7 cells, somBiPOLES n = 9 cells, one-way ANOVA, exact P-values are shown). The data presented in this figure and details on the statistical analysis are provided in the Source Data file.

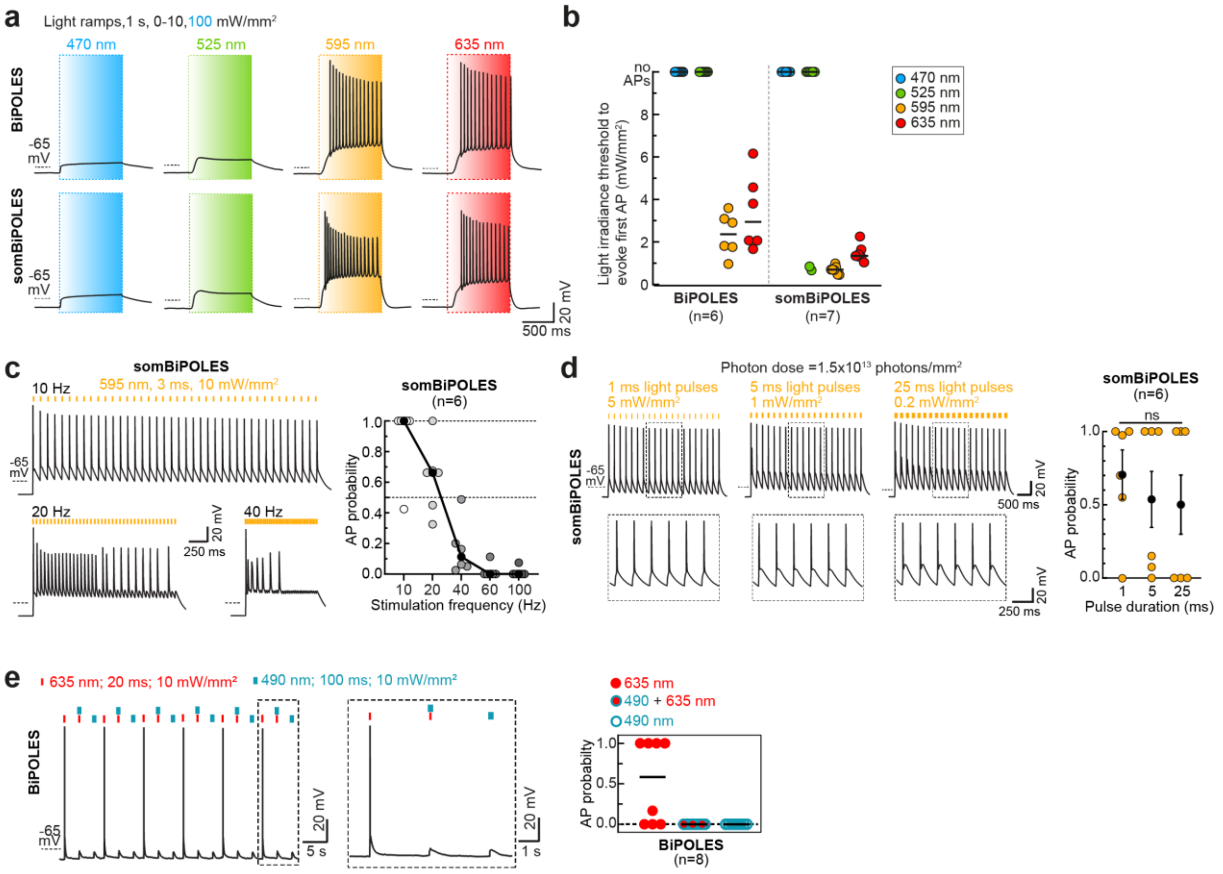

Supplementary Fig. 7. Optical spiking parameters for BiPOLES and somBiPOLES. (a,b) Spectral quantification of action potential threshold for BiPOLES and somBiPOLES. **(a)** Representative membrane voltage traces measured in BiPOLES- (top), or somBiPOLES-expressing CA1 pyramidal neurons (bottom). In IC experiments, light ramps of different wavelengths were applied as indicated. The irradiance was ramped linearly from 0 to 10 mW mm^{-2} over 1 s, except for 470-nm ramps, which were ranging to 100 mW mm^{-2} to rule out the possibility that high-intensity blue light might still evoke action potentials. **(b)** Quantification of the irradiance threshold at which the first action potential was evoked. 470-nm light up to 100 mW mm^{-2} did not evoke action potentials in BiPOLES or somBiPOLES-expressing cells. The irradiance threshold for 595 and 635 nm illumination was lower in somBiPOLES-expressing cells compared to BiPOLES-expressing cells indicating higher light sensitivity in the former (black horizontal lines: medians, $n_{BiPOLES}$ = 6 cells, $n_{somBiPOLES}$ =7 cells). somBiPOLES data for 470 and 595 nm are the same as in Fig. 3d. **(c)** Left: membrane voltage traces at different light-pulse frequencies in CA1 cells expressing somBiPOLES. APs were triggered by 40 pulses (λ = 595 nm, pulse width = 3 ms, 10 mW mm^{-2}). Right: Quantification of AP probability at increasing stimulation frequencies (from 10 to 100 Hz, black circles: medians, n = 6 cells). To determine AP probability, the number of light-triggered APs was divided by the total number of light pulses. **(d)** Left: membrane voltage traces at different light-pulse widths (1, 5 and 25 ms) and irradiances (5, 1, and 0.2 mW mm^{-2}, respectively). In all conditions the photon dose was kept constant at 1.5x10^{13} photons/mm^2. Magnified views of the traces are shown below. Note the different shapes of the sub-threshold membrane voltages evoked by the respective combination of parameters. Right: Quantification of AP probability at indicated light stimulation condition (black circles: mean ± SEM, n = 6 cells). **(e)** All-optical excitation and inhibition with BiPOLES. Current-clamp characterization of bidirectional optical spiking-control with BiPOLES. Left: Voltage traces showing red-light-evoked action potentials (APs), which were blocked by a concomitant blue light pulse. Right: quantification of AP probability under indicated conditions (black horizontal lines: medians, n = 8 cells). The data presented in this figure and details on the statistical analysis are provided in the Source Data file.

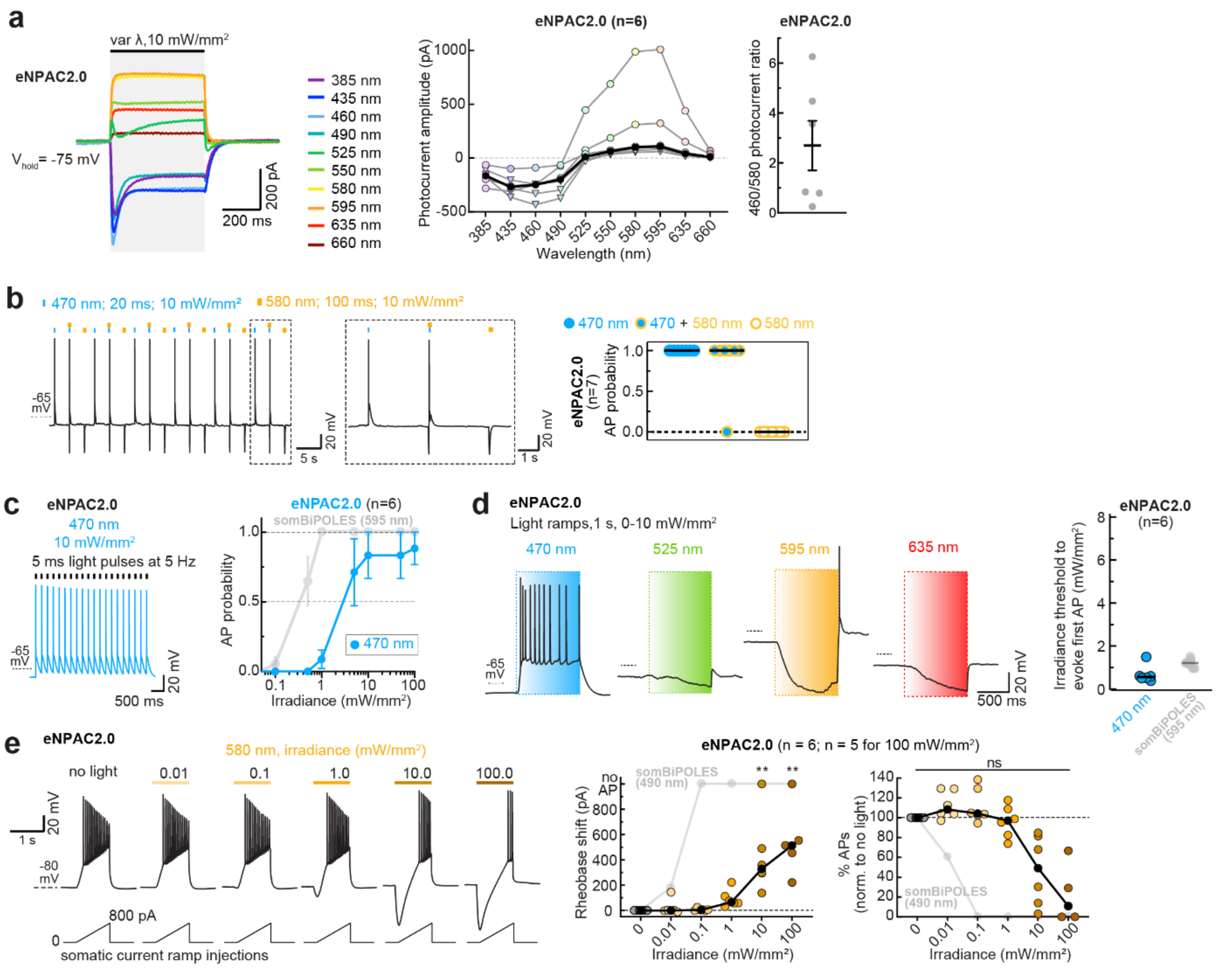

Supplementary Fig. 8. Characterization of bidirectional optogenetic manipulation of neuronal activity with eNPAC2.0. (a) Left: Representative eNPAC2.0 photocurrent traces in CA1 pyramidal neurons upon illumination with different wavelengths and equal photon flux at a membrane voltage of -75 mV. Middle: Quantification of photocurrent amplitude along the spectrum (black circles: medians, colored circles: photocurrents elicited by an irradiance of 10 mW mm^{-2}, colored triangles: photocurrents elicited by an irradiance of 1 mW mm^{-2}, n = 6 cells). Similar to HEK-cell measurements (see Supplementary Fig. 2b), inward and outward photocurrents were evoked with blue and orange light, respectively, indicative of independently evoked ChR2(H134R)- and eNpHR3.0-photocurrents. Right: Quantification of the ratio of excitatory (460 nm) over inhibitory (580 nm) photocurrents (black line: mean ± SEM, n = 6 cells). Note that this ratio is more scattered compared to BiPOLES (see Supplementary Fig. 3c), indicating variability in the stoichiometry of excitatory and inhibitory opsins between cells. This is likely explained by the different expression strategies for eNPAC2.0 (bi-cistronic, p2A construct) and BiPOLES (fusion protein and 1:1 stoichiometric expression of both tandem partners). **(b)** Characterization of all-optical spiking and silencing with eNPAC2.0. Left: Voltage traces showing blue-light-evoked APs, which, under the indicated conditions, could not be blocked by stimulation of eNpHR3.0 with a concomitant yellow light pulse. Yellow light alone led to a hyperpolarization of membrane voltage, indicating chloride loading of the cell by eNpHR3.0. Right: quantification of AP probability under indicated conditions (black horizontal lines: medians, n = 7 cells). **(c)** Left: Example traces of voltage clamp recordings of eNPAC2.0 to determine light-evoked AP probability with 470 nm. Right: quantification of light-mediated AP probability at indicated irradiances (symbols represent mean ± SEM, n = 6 cells). Note that even at an irradiance of 100 mW mm^{-2} not all cells achieved 100% spiking probability. This contrasts with CA1 cells expressing somBiPOLES or Chrimson alone, where 100% spiking probability is achieved with 595-nm light (their peak activation wavelength) at irradiances around 1 mW mm^{-2} (see Fig. 3b,c). **(d)** Spectral quantification of the irradiance threshold for AP generation with

eNPAC2.0. Left: Representative membrane voltage traces during light ramps at indicated wavelengths with irradiance increasing linearly from 0 to 10 mW mm^{-2}. Note that a rebound spike was triggered after applying a 595-nm light ramp. Right: Quantification of the irradiance threshold at which the first AP was evoked (black horizontal lines: medians, n = 6 cells). **(e)** eNPAC2.0 mediates neuronal membrane voltage hyperpolarization upon illumination with yellow light. Left: Current ramps (from 0–100 to 0–900 pA) were injected into eNPAC2.0-expressing CA1 pyramidal cells to induce APs during illumination with yellow light at indicated intensities (from 0.01 to 100 mW mm^{-2}). Right: Quantification of the rheobase shift and the relative change in the number of ramp-evoked action potentials. The injected current at the time of the first action potential was defined as the rheobase. Illumination with 580 nm light of increasing intensities activated eNpHR3.0-mediated Cl^- pumping, which strongly hyperpolarized the membrane voltage, shifting the rheobase to higher values and shunting APs. Note that the ability of eNPAC2.0 to silence neurons is smaller compared to somBiPOLES (see Fig. 3g). eNPAC2.0 required 2 orders of magnitude higher irradiance to achieve a significant shift of the rheobase (black circles: medians, n = 6, one-way Friedman test, *p < 0.05, **p < 0.01, ***p < 0.001). Grey symbols and lines in (c), (d) and (e) are somBiPOLES values from Fig. 3 plotted for comparison. The data presented in this figure and details on the statistical analysis are provided in the Source Data file.

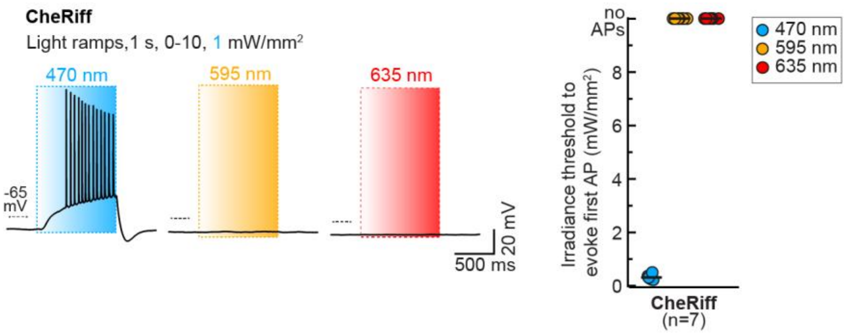

Supplementary Fig. 9. CheRiff exhibits optical excitation restricted to the blue spectrum. (a) Left: Representative membrane voltage traces measured in CheRiff-expressing CA1 pyramidal neurons. In IC experiments, light ramps of different wavelengths were applied as indicated. Light was ramped linearly from 0 to 10 mW mm^{-2} over 1 s. 470-nm ramps were ranging only up to 1 mW mm^{-2}, which was already sufficient to evoke APs. Right: Quantification of the irradiance threshold at which the first AP was evoked. Orange/red light up to 10 mW mm^{-2} did not evoke action potentials in CheRiff-expressing cells (black horizontal lines: medians, n = 7 cells). The data presented in this figure are provided in the Source Data file.

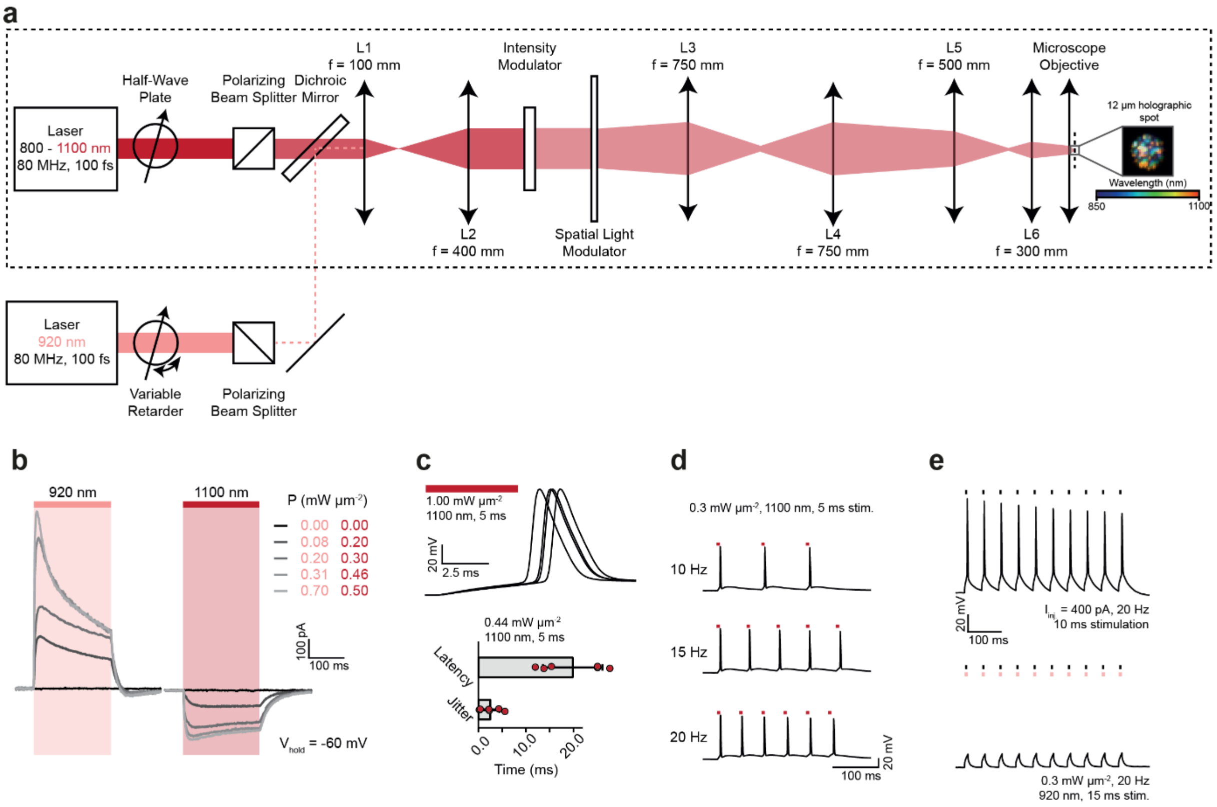

Supplementary Fig. 10. Design of the dual-laser 2-photon holography setup. (a) A schematic diagram of the experimental setup used for two-photon photo-stimulation and inhibition using holography. The optical path indicated by the black, dashed rectangle was used to acquire all data presented in Fig. 5. The system was aligned at the central wavelength (980 nm), but holograms at all wavelengths were co-aligned laterally and axially as demonstrated in the inset. Double-headed arrows are used to illustrate lenses, denoted by L, with focal lengths denoted by f. The reflective Spatial Light Modulator (SLM) is shown as transmissive for illustrative purposes. The photoinhibition beam (920 nm) was combined with the beam from the tunable laser using a dichroic mirror. The precise details of each optical component can be found in the main text. **(b)** Representative photocurrent traces at a range of different average power densities, obtained by continuous 200 ms illumination of 920 and 1100 nm at a holding potential of -60 mV. **(c)** Top: Representative traces of photo-evoked action potentials. Bottom: Mean latency and jitter calculated as the average of 5 trials in different neurons. Error bars represent the standard deviation across trials. **(d)** Representative photo evoked trains of action potentials under 1100-nm illumination at different stimulation frequencies. **(e)** Demonstration of precise elimination of single action potentials using short (15 ms) pulses of 920 nm light. Upper trace (control): electrically induced 20 Hz spike train by 10 ms injection of 400 pA current. Lower trace: suppression of electrically induced action potentials by co-incident illumination of 15 ms pulses of 920 nm light. The data presented in this figure are provided in the Source Data file.

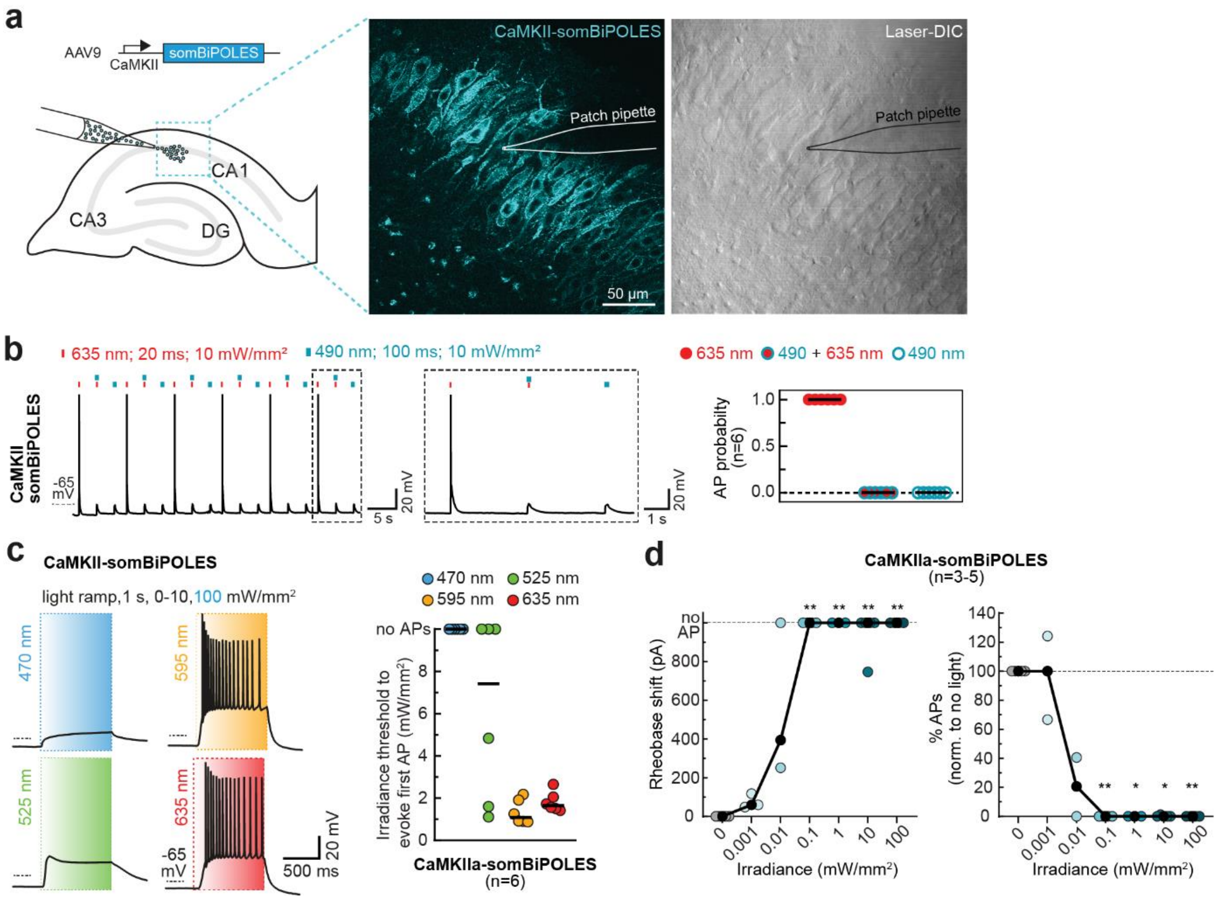

Supplementary Fig. 11. Virally expressed CaMKII-somBiPOLES enables bidirectional control of activity in projection neurons. (a) Viral transduction of CaMKII-somBiPOLES in hippocampal organotypic slice cultures. Right: Single-plane 2-photon fluorescence (cyan) and laser-DIC (gray) example images showing expression of somBiPOLES in pyramidal cells of *stratum pyramidale* and cellular morphology, respectively. The position of the patch pipette is depicted by a drawing of its outline. **(b)** IC characterization of bidirectional optical spiking-control with CaMKII-somBiPOLES. Left: Voltage traces showing red-light-evoked APs, which were blocked by a concomitant blue-light pulse. Blue light alone did not trigger APs. Right: quantification of AP probability under indicated conditions (black horizontal lines: medians, n = 6 cells). **(c)** Left: Representative membrane voltage traces measured in CaMKII-somBiPOLES-expressing pyramidal neurons. In IC experiments, light ramps were applied as indicated. Light was ramped linearly from 0 to 10 mW mm^{-2} over 1 s, except for 470 nm ramps, which were ranging to 100 mW mm^{-2} to rule out the possibility that high-irradiance blue light might still evoke APs. Right: Quantification of the irradiance threshold at which the first AP was evoked (black horizontal lines: medians, n = 6 cells). **(d)** Quantification of CaMKII-somBiPOLES-mediated neuronal silencing. Current ramps (from 0–100 to 0–900 pA) were injected into CaMKII-somBiPOLES-expressing cells to induce APs. The injected current at the time of the first AP was defined as the rheobase. Illumination with blue light of increasing irradiance (from 0.001 to 100 mW mm^{-2}) activated *Gt*ACR2-mediated Cl^- currents shifting the rheobase to higher values (black circles: medians, n = 5 cells (in 3 cells rheobase shift and %APs were measured for all light irradiances, in 1 cell for 0.0 0.1, 10 and 100 mW mm^{-2}; and in 1 cell only for 0.0 and 0.1 mW mm^{-2}) , one-way Kruskal-Wallis test, **$p < 0.01$, **$p < 0.001$). The data presented in this figure and details on the statistical analysis are provided in the Source Data file.

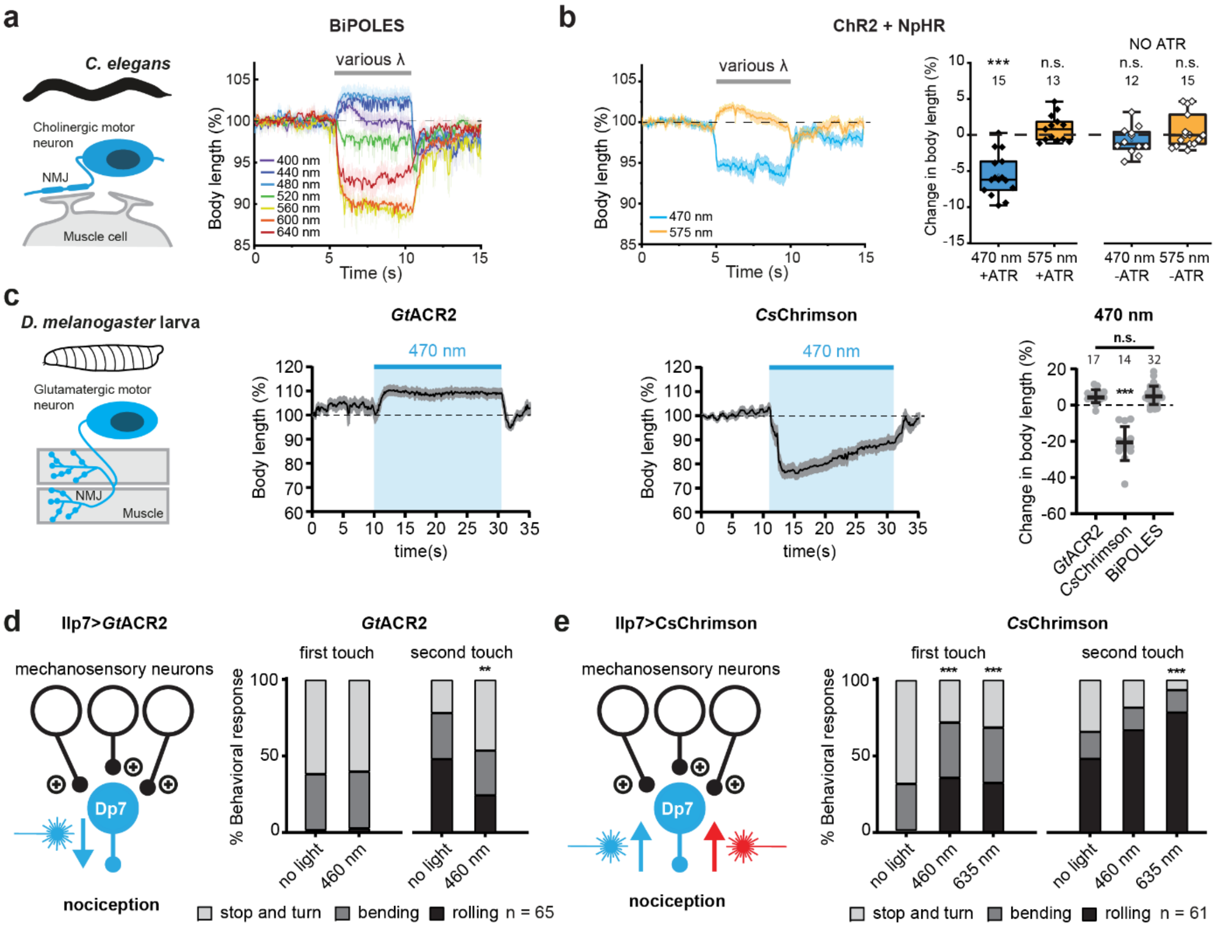

Supplementary Fig. 12. BiPOLES and controls in *C. elegans* and *D. melanogaster*. (a) Precise timing of bidirectional control of cholinergic motor neurons in *C. elegans*. Temporal dynamics of relative changes in body length upon illumination with light at wavelengths ranging from 400 to 640 nm in *C. elegans* expressing BiPOLES in cholinergic motor neurons (mean ± SEM, 1.1 mW mm^{-2}, 400 nm, n = 9; 440 nm, n = 12; 480 nm, n = 10; 520 nm, n = 12; 560 nm, n= 9; 600 nm, n = 13; 640 nm, n = 11). **(b)** Left: temporal dynamics of relative changes in body length upon illumination with light at 470 and 575 nm in *C. elegans* expressing ChR2(HR) and NpHR in cholinergic motor neurons (mean ± SEM, 1.1 mW mm^{-2}, 400 nm, n = 9; 440 nm, n = 12; 480 nm, n = 10; 520 nm, n = 12; 560 nm, n= 9; 600 nm, n = 13; 640 nm, n = 11). Right: quantification of maximal change in body length (Box: median, 1st – 3rd quartile, whiskers: 1.5x inter quartile range, ***p < 0.0001, paired, two-sided t-test, p values of comparisons of the stimulated condition (seconds 6-9 against the non-stimulated condition (seconds 0-4): 470 nm with ATR (n = 15): 6.4E-8, 575 nm with ATR (n = 13): 0.11, 470 nm without ATR (n = 12): 0.21, 575 nm without ATR (n = 15): 0.73). Note that NpHR stimulation did not lead to significant body relaxation. **(c)** *Gt*ACR2 or *Cs*Chrimson expressed alone in glutamatergic neurons of *D. melanogaster* larvae (*OK371-Gal4>UAS-GtACR2* or *UAS-CsChrimson*) result in opposite responses upon blue light stimulation. Schematic of *Gt*ACR2- or *Cs*Chrimson-expressing glutamatergic motor neuron innervating muscle fibers. Middle: Temporal dynamics of relative changes in body length upon illumination with 470 nm light (mean ± SEM, 17 µW/mm^2, n = 32). Right: Quantification of maximal change in body length (mean ± SEM, *Gt*ACR2, n = 17; *Cs*Chrimson, n = 14; BiPOLES, n = 32, ***p < 0.0001). Note that similar to BiPOLES, blue light illumination of animals expressing *Gt*ACR2 alone leads to body relaxation (BiPOLES dataset from Fig. 6d). In contrast, *Cs*Chrimson alone induces body constriction under blue light. **(d)** *Gt*ACR2 expression in Dp7 neurons in *Drosophila* larvae (*Ilp7-Gal4>UAS-GtACR2*) and behavioral response after the first and second mechanical stimulus under blue light (470 nm) compared to no light shows comparable inhibition of rolling as BiPOLES. n = 60 **p = 0.0057, X^2-test. **(e)** *Cs*Chrimson expression in Dp7 neurons (*Ilp7-Gal4>UAS-CsChrimson*) and behavioral response after the first and second mechanical stimulus under blue light (470 nm, 1.7 µmW mm^{-2}) or red light (635 nm, 2.5 µW/mm^2) illumination compared to no light. Note that unlike with BiPOLES, blue light and red light

increased rolling responses with *Cs*Chrimson. n = 61, ***p < 0.0001, X^2-test. The data presented in this figure and details on the statistical analysis are provided in the Source Data file.

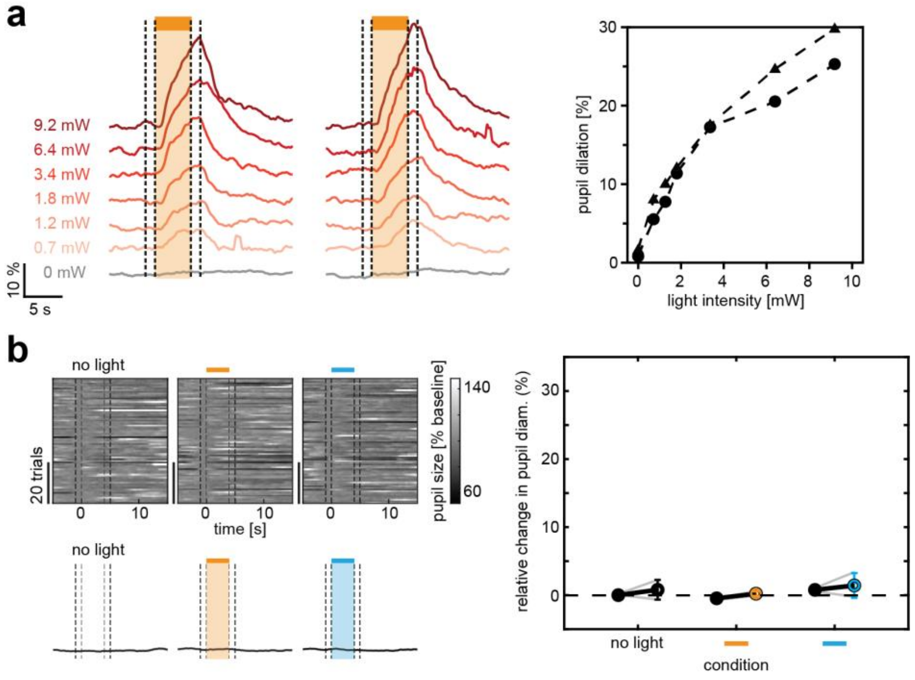

Supplementary Fig. 13. somBiPOLES controls in LC neurons. (a) The magnitude of pupil dilation scales with the red-light irradiance. Quantification of normalized pupil size in two animals under indicated light powers per fiber (594 nm). Dashed lines show regions used for quantification in the plot on the right. **(b)** Pupil dilation is not altered by light applied to the LC in fiber-implanted, non-injected wild-type animals. Quantification of normalized pupil size in one wild-type animal under various stimulation conditions as indicated. Orange and blue bars indicate time of illumination with 594 (orange) and 473 nm (blue), respectively. Top left: single trials. Bottom left: mean ± SEM. Dashed lines show time points used for quantification in the plot on the right. Right: quantification of relative pupil size (n = 3 mice; One-way analysis of variance; F = 0.01, p = 0.99). The data presented in this figure and details on the statistical analysis are provided in the Source Data file.

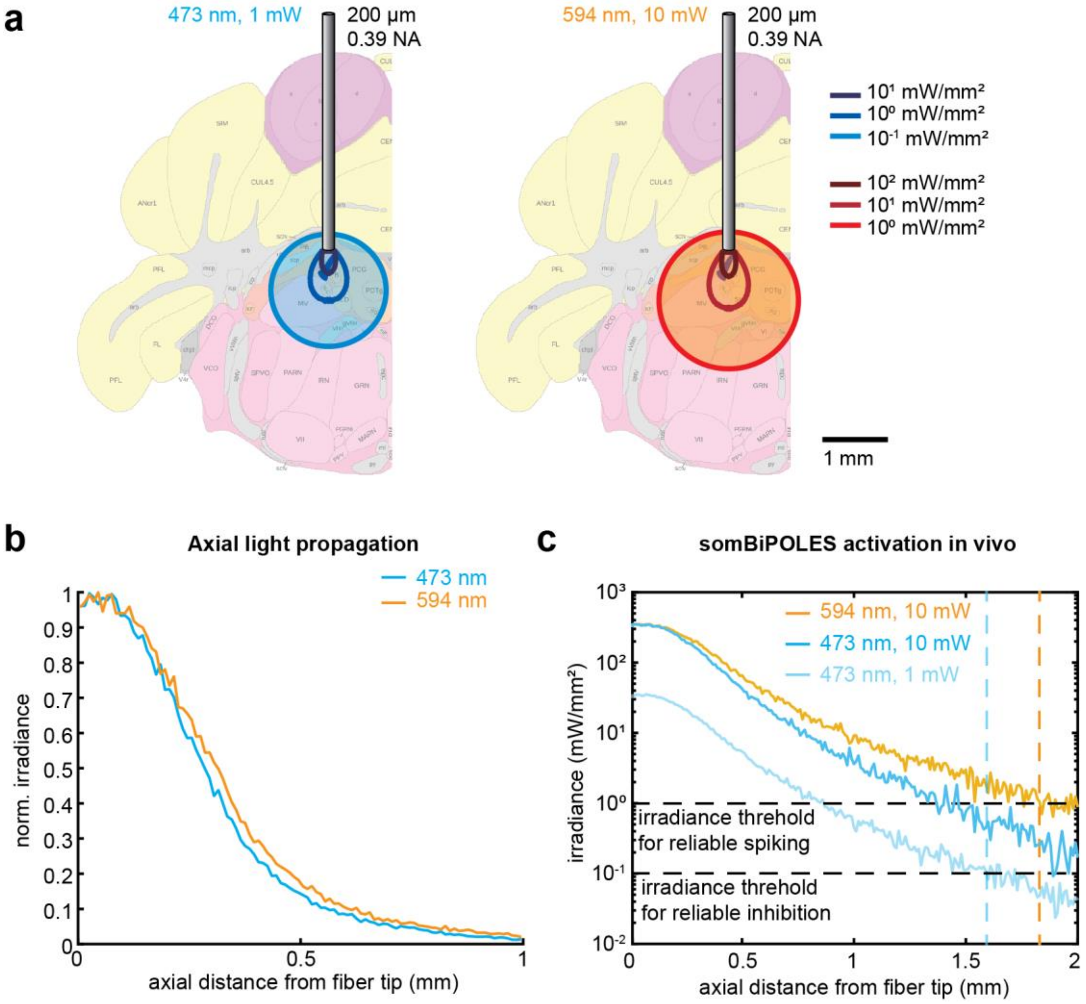

Supplementary Fig. 14. Monte-Carlo simulation of light propagation in the mouse brain to estimate somBiPOLES performance in vivo. **(a)** Simulation of light propagation (473 nm, left and 594 nm, right) from the tip of an optical fiber implanted above Locus Coeruleus in the mouse brain. Contour lines indicate interval of one log unit. **(b)** Simulation of the axial irradiance perpendicular to the fiber tip. Note the minimal differences in attenuation of blue light vs. orange light. **(c)** Estimation of reliable somBiPOLES performance under indicated light conditions. Reliable spiking of neurons can be achieved up to ~1.8 mm away from the fiber tip with 10 mW of 594 nm light. Similarly, efficient shunting of neuronal activity is achieved up to ~1.6 mm from the fiber tip with 1 mW of 473 nm light. The blue and orange irradiance thresholds required for reliable silencing and spiking are derived from Fig. 3. The data presented in this figure are provided in the Source Data file.

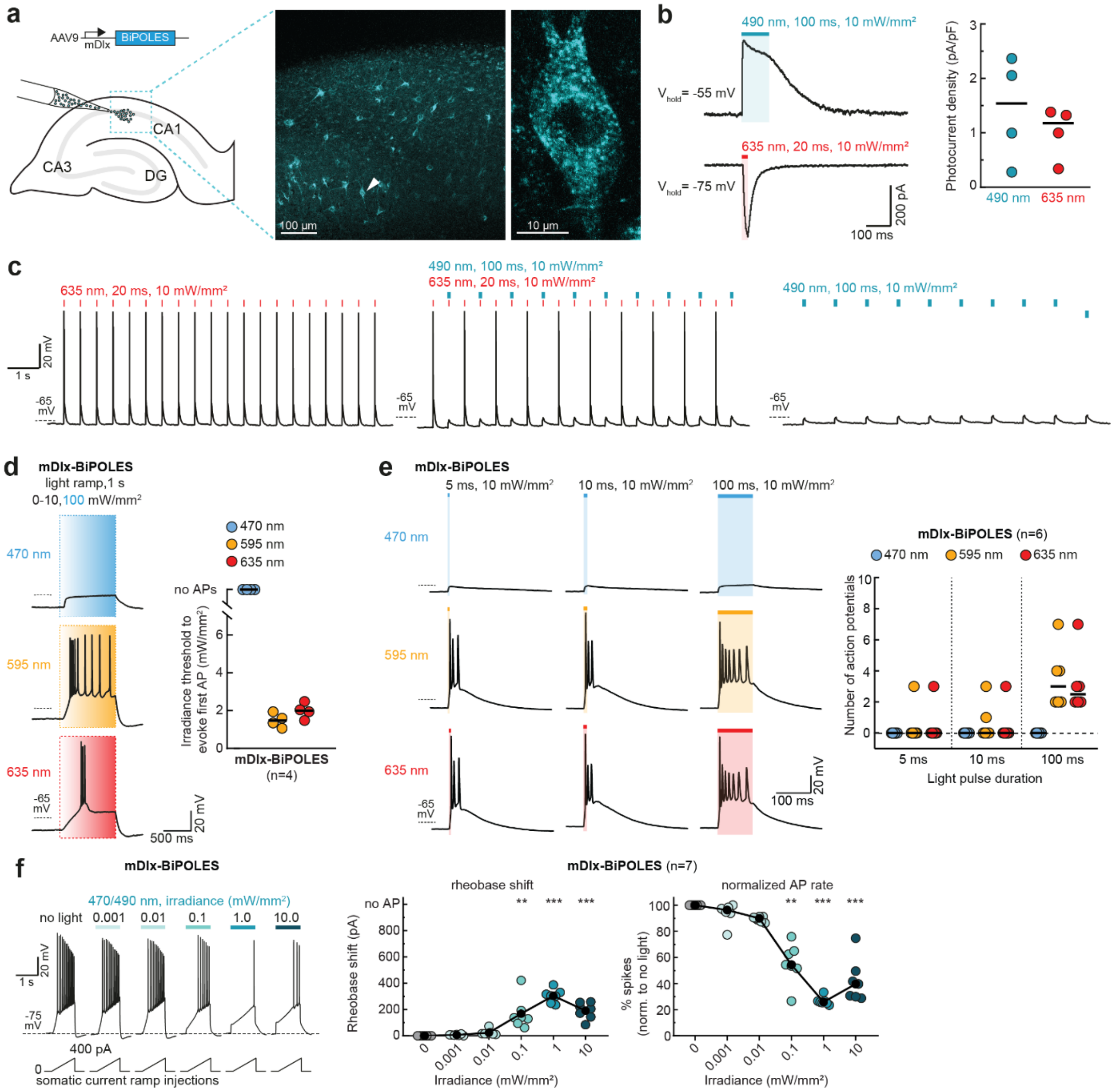

Supplementary Fig. 15. Virally expressed mDlx-BiPOLES enables bidirectional control of GABAergic neuronal activity. (a) Viral transduction of mDlx-BiPOLES in hippocampal organotypic slice cultures. Right: Representative maximum-intensity projection image of a 2-photon stack showing expression of BiPOLES in GABAergic neurons in CA1. Magnified view of a single neuron indicated by white arrowhead is shown on the right. **(b)** Left: Representative photocurrent traces measured in an mDlx-BiPOLES-expressing CA1 GABAergic neuron. Photocurrents evoked by a 490 nm light pulse (100 ms, 10 mW mm^{-2}) were recorded at a membrane voltage of -55 mV and photocurrents evoked by a 635 nm light pulse (20 ms, 10 mW mm^{-2}) were recorded at a membrane voltage of -75 mV. Right: Quantification of photocurrent densities evoked under the indicated conditions (black horizontal lines: medians, n = 4 cells). **(c)** IC characterization of bidirectional optical spiking-control with mDlx-BiPOLES. Voltage traces showing red-light-evoked APs (left), which were blocked by a concomitant blue-light pulse (middle). Blue light alone did not trigger APs (right). **(d)** Left: Representative IC membrane voltage traces measured in mDlx-BiPOLES-expressing neurons. In IC experiments, light ramps were applied as indicated. Irradiance was ramped linearly over 1 s from 0 to 10 mW mm^{-2} or to 100 mW mm^{-2} for 470 nm to rule out the possibility that high-irradiance blue light might still evoke action potentials. Right: Quantification of the irradiance threshold at which the first AP was evoked (black horizontal lines: medians, n = 4 cells) 470-nm light up to 100 mW mm^{-2} did not evoke APs in mDlx-BiPOLES-expressing cells, while 595 and 635 nm light evoked APs at irradiance levels comparable to pyramidal cells expressing BiPOLES (see Supplementary Fig. 7a,b). **(e)** Extended duration of illumination increased the

probability and number of action potentials. Left: Representative IC membrane voltage traces measured in mDlx-BiPOLES-expressing neurons illuminated as indicated. Right: quantification of the number of action potentials evoked by the different illumination protocols (black horizontal lines: medians, n = 6 cells). **(f)** Quantification of mDlx-BiPOLES-mediated neuronal silencing. Current ramps (from 0–100 to 0–900 pA) were injected into mDlx-BiPOLES-expressing cells to induce action potentials. The injected current at the time of the first action potential was defined as the rheobase. Illumination with blue light of increasing irradiance (from 0.001 to 10.0 mW mm^{-2}) activated *Gt*ACR2-mediated Cl^- currents shifting the rheobase to higher values. Middle: Quantification of the rheobase shift at different light intensities. Right: Relative change in the number of ramp-evoked action potentials upon illumination with blue light at indicated irradiance values (black circles: medians, n = 7 cells, one-way Friedman test, **p < 0.01, ***p < 0.001). The data presented in this figure and details on the statistical analysis are provided in the Source Data file.
