# Supplementary material for: BiPOLES is an optogenetic tool developed for bidirectional dual-color control of neurons
Source: Nat Commun. 2021 Jul 26;12:4527. doi: 10.1038/s41467-021-24759-5 (PMC8313717; doi:10.1038/s41467-021-24759-5)
Supplement: Supplementary file 2 — Reporting Summary [file 41467_2021_24759_MOESM2_ESM.pdf]

nature research

Corresponding author(s): J. Simon Wiegert

Last updated by author(s): Jun 23, 2021

Reporting Summary

Nature Research wishes to improve the reproducibility of the work that we publish. This form provides structure for consistency and transparency in reporting. For further information on Nature Research policies, see our Editorial Policies and the Editorial Policy Checklist.

Statistics

For all statistical analyses, confirm that the following items are present in the figure legend, table legend, main text, or Methods section.

n/a	Confirmed	
☐	☒	The exact sample size (n) for each experimental group/condition, given as a discrete number and unit of measurement
☐	☒	A statement on whether measurements were taken from distinct samples or whether the same sample was measured repeatedly
☐	☒	The statistical test(s) used AND whether they are one- or two-sided<br>*Only common tests should be described solely by name; describe more complex techniques in the Methods section.*
☒	☐	A description of all covariates tested
☐	☒	A description of any assumptions or corrections, such as tests of normality and adjustment for multiple comparisons
☐	☒	A full description of the statistical parameters including central tendency (e.g. means) or other basic estimates (e.g. regression coefficient) AND variation (e.g. standard deviation) or associated estimates of uncertainty (e.g. confidence intervals)
☐	☒	For null hypothesis testing, the test statistic (e.g. F, t, r) with confidence intervals, effect sizes, degrees of freedom and P value noted<br>*Give P values as exact values whenever suitable.*
☒	☐	For Bayesian analysis, information on the choice of priors and Markov chain Monte Carlo settings
☒	☐	For hierarchical and complex designs, identification of the appropriate level for tests and full reporting of outcomes
☒	☐	Estimates of effect sizes (e.g. Cohen's d, Pearson's r), indicating how they were calculated

Our web collection on statistics for biologists contains articles on many of the points above.

Software and code

Policy information about availability of computer code

Data collection	pCLAMP V11, Molecular Devices; SutterPatch V2, Sutter Instrument; ScanImage 2017b, Vidrio Technologies; MATLAB 2016b, 2019b, Mathworks; IC Capture, The Imaging Source, Arduino 1.0.6, ZEISS ZEN Software, FIMTrack; Previously published, custom-written code was used for two-photon holography. The source of the original code is indicated by the respective references in the manuscript.
Data analysis	pCLAMP V11, Molecular Devices; SutterPatch V2, Sutter Instrument; MATLAB 2016b, 2019b, Mathworks, ImageJ v1.51t, OriginLab 2018, FIMTrack; Previously published, custom-written code was used for analysis of C. elegans body length, pupil size in mice, and electrophysiology data from ferrets. The source of the original code is indicated by the respective references in the manuscript.

For manuscripts utilizing custom algorithms or software that are central to the research but not yet described in published literature, software must be made available to editors and reviewers. We strongly encourage code deposition in a community repository (e.g. GitHub). See the Nature Research guidelines for submitting code & software for further information.

Data

Policy information about availability of data

All manuscripts must include a data availability statement. This statement should provide the following information, where applicable:

- Accession codes, unique identifiers, or web links for publicly available datasets
- A list of figures that have associated raw data
- A description of any restrictions on data availability

Source data are provided with this paper. All data generated in this study are provided in the Source Data file.

Field-specific reporting

Please select the one below that is the best fit for your research. If you are not sure, read the appropriate sections before making your selection.

- [x] Life sciences
- [ ] Behavioural & social sciences
- [ ] Ecological, evolutionary & environmental sciences

For a reference copy of the document with all sections, see nature.com/documents/nr-reporting-summary-flat.pdf

Life sciences study design

All studies must disclose on these points even when the disclosure is negative.

Sample size

Sample-size calculations were not performed as the effect size was not known before the study. However, sample sizes for the different experiments presented in this study were matched to published experiments that used similar methodology, model systems and manipulations.
HEK cell experiments: DOI: 10.1038/s41467-018-06421-9, DOI: 10.1126/science.1249375
Hippocampal slice cultures: DOI: 10.1126/science.1249375, 10.1038/s41467-018-06421-9, DOI: 10.1038/s41598-017-14330-y, DOI: 10.1038/srep14807
C. elegans experiments: DOI: 10.1016/j.cell.2018.09.026, DOI: 10.1038/nature05744, DOI: 10.1016/j.cub.2005.11.032
D. melanogaster experiments: DOI: 10.1038/s41598-017-14330-y, DOI: 10.1038/nn.4580
Mouse in vivo experiments: DOI: 10.1038/s41593-018-0305-z
Ferret ephys experiments: DOI: 10.1126/sciadv.aar7633

Data exclusions

In patch-clamp experiments, we excluded recordings that were not stable throughout the duration of the experiment or where a proper recording configuration could not be achieved. HEK-cell recordings with a membrane resistance below 500 MOhm or an access resistance higher than 10 MOhm were excluded as reported in the Methods section. Recordings from hippocampal neurons with a membrane resistance below 50 MOhm and a series resistance over 25 MOhm were not analyzed. For experiments in C. elegans, we excluded videos of animals showing coiling behavior, since this restricts recognition by the evaluation software. For recordings in ferret visual cortex, we only considered contacts that showed a significant modulation upon visual stimulation.

Replication

All experiments were replicated multiple times in biologically independent samples (HEK cells, slices, worms, fly larvae, mice, ferrets). The precise n numbers are reported in the manuscript. In addition, we performed a large number of similar experiments with only slight modifications, such as expression of the same BiPOLES version from different promoters or using different transduction methods for neuronal expression (Supplemental figures 11, 15). These experiments had comparable outcomes. Moreover, a number of research groups, not involved in this study, independently replicated the main findings of this paper already.

Randomization

In all experiments, the spectral order of light delivery, or the order of light intensities were shuffled. In experiments, where cells were held at different membrane voltages, this parameter was also shuffled. For in vivo experiments, the conditions were randomized and trials with different conditions were interleaved. Fluorescent HEK cells were randomly selected for patch-clamp experiments. Hippocampal slice cultures were randomly chosen for single-cell electroporation or virus transduction. Transgene expressing cells were randomly selected for patch-clamp experiments, provided they showed intact morphology in the DIC image. For experiments with C. elegans and D. melanogaster, transgenic animals expressing the transgene of interest were randomly collected from a larger stock. Mice and ferrets were not randomized since each animal expressed the same transgenes. Randomization was rather performed on the stimulus protocols as described above.

Blinding

Data analysis of HEK-cell recordings and neuronal recordings was not done blinded, because opsin-mediated effects were apparent in the recordings and thereby revealed the condition. However, pre-established, semi-automatic analysis pipelines were used that did not permit dismissal of any data points or experiments by the analyst based on the analysis result. Analysis of C. elegans and D. melanogaster experiments was done blinded to the light condition. Analysis of experiments in mice and ferrets was done with automatized routines and did not require blinding.

Reporting for specific materials, systems and methods

We require information from authors about some types of materials, experimental systems and methods used in many studies. Here, indicate whether each material, system or method listed is relevant to your study. If you are not sure if a list item applies to your research, read the appropriate section before selecting a response.

Materials & experimental systems

n/a	Involved in the study	
	[x]	Antibodies
	[x]	Eukaryotic cell lines
[x]		Palaeontology and archaeology
	[x]	Animals and other organisms
[x]		Human research participants
[x]		Clinical data
[x]		Dual use research of concern

Methods

n/a	Involved in the study	
[x]		ChIP-seq
[x]		Flow cytometry
[x]		MRI-based neuroimaging

Antibodies

Antibodies used

chicken, anti-GFP, Invitrogen, A10262, Lot 1972783, 1:1000; goat, anti-chicken Alexa-488, Invitrogen; A11039, Lot 2079383, 1:1000

Validation

anti-GFP Antibody (A10262) was verified by Relative expression to ensure that the antibody binds to the antigen stated.
Antibody specificity was demonstrated by detection of different targets fused to GFP tag in transiently transfected lysates tested. Relative detection of GFP tag was observed across different proteins fused with GFP in H3-GFP (Lane 3-5) and p65-GFP (Lane 6). GFP-variant, YFP is also being detected in His-p65-YFP lysate (Lane 7), using Anti-GFP Polyclonal Antibody (Product # A10262) in Western Blot. Relative expression validation info.

anti-chicken IgY (H+L) Secondary Antibody (A11039) was tested in IHC as follows:
A section of mouse intestine was stained with a combination of fluorescent stains. Fibronectin, an extracellular matrix adhesion molecule, was labeled using a chicken primary antibody against fibronectin and visualized using green-fluorescent Alexa Fluor® 488 Goat Anti-Chicken IgG antibody (Product # A-11039). The filamentous actin (F-actin) prevalent in the brush border was stained with red-fluorescent Alexa Fluor® 568 phalloidin (Product # A12380). Finally, the nuclei were stained with DAPI (D1306, D3571, D21490).

Eukaryotic cell lines

Policy information about cell lines

Cell line source(s)

HEK293

Authentication

HEK293 was purchased from DSMZ (Leibniz Institute DSMZ-German Collection of Microorganisms and Cell Cultures)

Mycoplasma contamination

not tested for HEK293

Commonly misidentified lines
(See ICLAC register)

n/a

Animals and other organisms

Policy information about studies involving animals; ARRIVE guidelines recommended for reporting animal research

Laboratory animals

Organotypic hippocampal slices were prepared from Wistar rats, C57Bl6J-mice (Janvier labs) or VIP-IRES-Cre mice (Jackson No. 031628) of both sexes at postnatal day 5-8; in vivo experiments were done with transgenic Caenorhabditis elegans: ZX2586 (wild type; zxEx1228[punc-17::GtACR2::mCerulean::bHK::Chrimson; pelt-2::GFP]); transgenic Drosophila melanogaster (OK371-Gal4, Ilp7-Gal4); adult TH-Cre mice (3-5 months) of both sexes (Jackson No. 008601); and adult female wild-type ferrets (Mustela putorius).

Wild animals

n/a

Field-collected samples

n/a

Ethics oversight

All procedures were performed in compliance with German law according and the guidelines of Directive 2010/63/EU. Protocols were approved by the Behörde für Gesundheit und Verbraucherschutz of the City of Hamburg.

Note that full information on the approval of the study protocol must also be provided in the manuscript.
