# Supplementary material for: BiPOLES is an optogenetic tool developed for bidirectional dual-color control of neurons
Source: Nat Commun. 2021 Jul 26;12:4527. doi: 10.1038/s41467-021-24759-5 (PMC8313717; doi:10.1038/s41467-021-24759-5)
Supplement: Supplementary file 5 — Description of Additional Supplementary Files [file 41467_2021_24759_MOESM5_ESM.pdf]

Title: Supplementary Data 1

Description: This document contains the nucleotide sequences encoding the various genes and constructs that were generated and used in this study. It also contains a list of primers used for cloning of new DNA constructs
